# Supplementary figures and images for: Deep learning tools and modeling to estimate the temporal expression of cell cycle proteins from 2D still images
Source: PLoS Comput Biol. 2022 Mar 14;18(3):e1009949. doi: 10.1371/journal.pcbi.1009949 (PMC8947602; doi:10.1371/journal.pcbi.1009949)

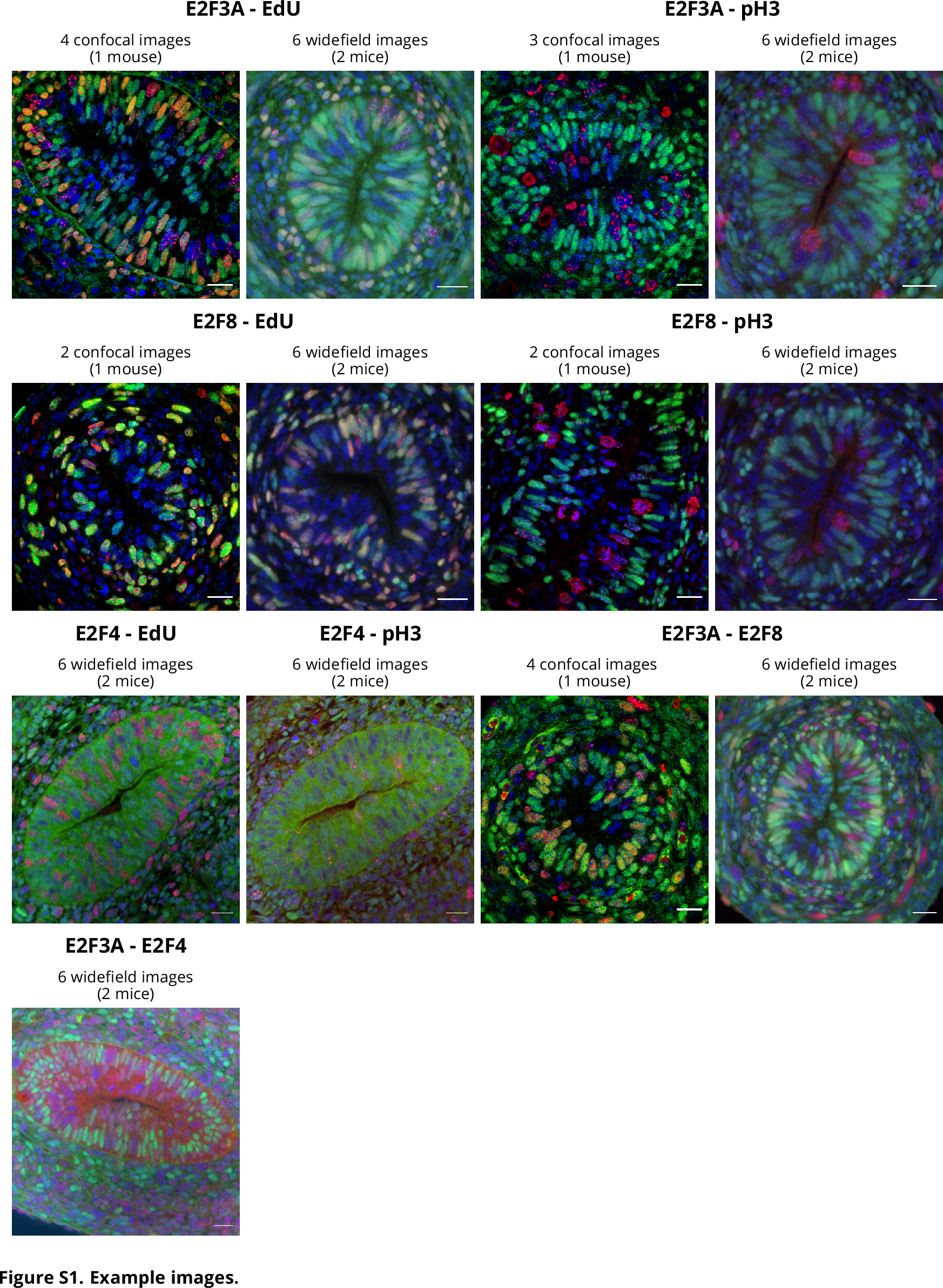

Supplement: S1 Fig — One example for each combination of markers and modality for fluorescence images used in the study. The combination of markers, number of images of each type, modality, and number of mice used in the study are shown on top of the images. Note the small size of the training set. Scale bar = 20μm. (TIF) [file pcbi.1009949.s001.tif]

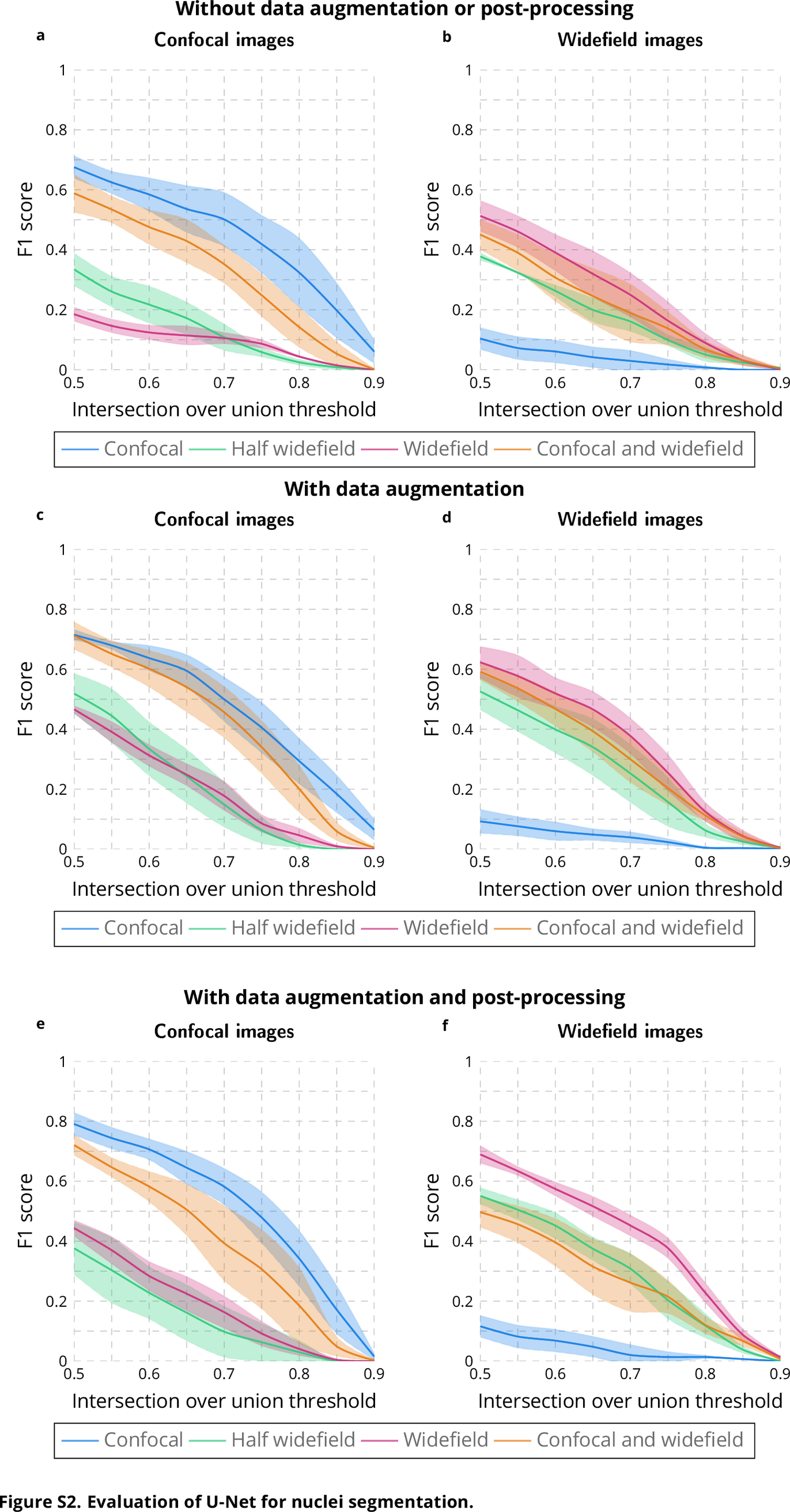

Supplement: S2 Fig — F1 score for multiple IoU thresholds obtained with the U-Net approach by trainig on confocal, half or all widefield images, and on confocal and widefield images without data augmentation or post-processing a-b, with data augmentation c-d, with data augmentation and corrected watershed postprocessing e-f. The lines correspond to the average F1 score while the areas represent the standard error. (TIF) [file pcbi.1009949.s002.tif]

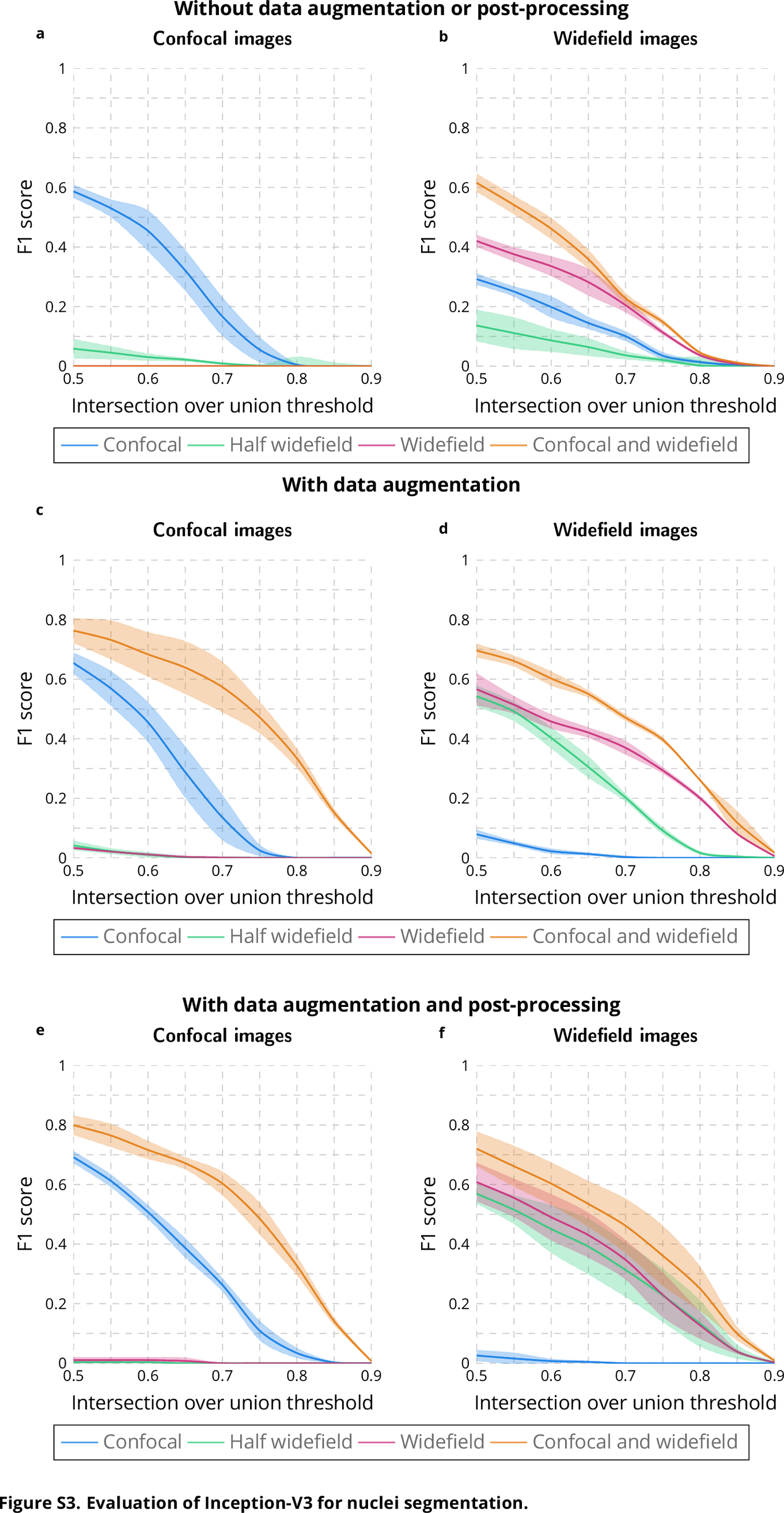

Supplement: S3 Fig — F1 score for multiple IoU thresholds obtained with the Inception-V3 approach by trainig on confocal, half or all widefield images, and on confocal and widefield images without data augmentation or post-processing a-b, with data augmentation c-d, with data augmentation and corrected watershed postprocessing e-f. The lines correspond to the average F1 score while the areas represent the standard error. (TIF) [file pcbi.1009949.s003.tif]

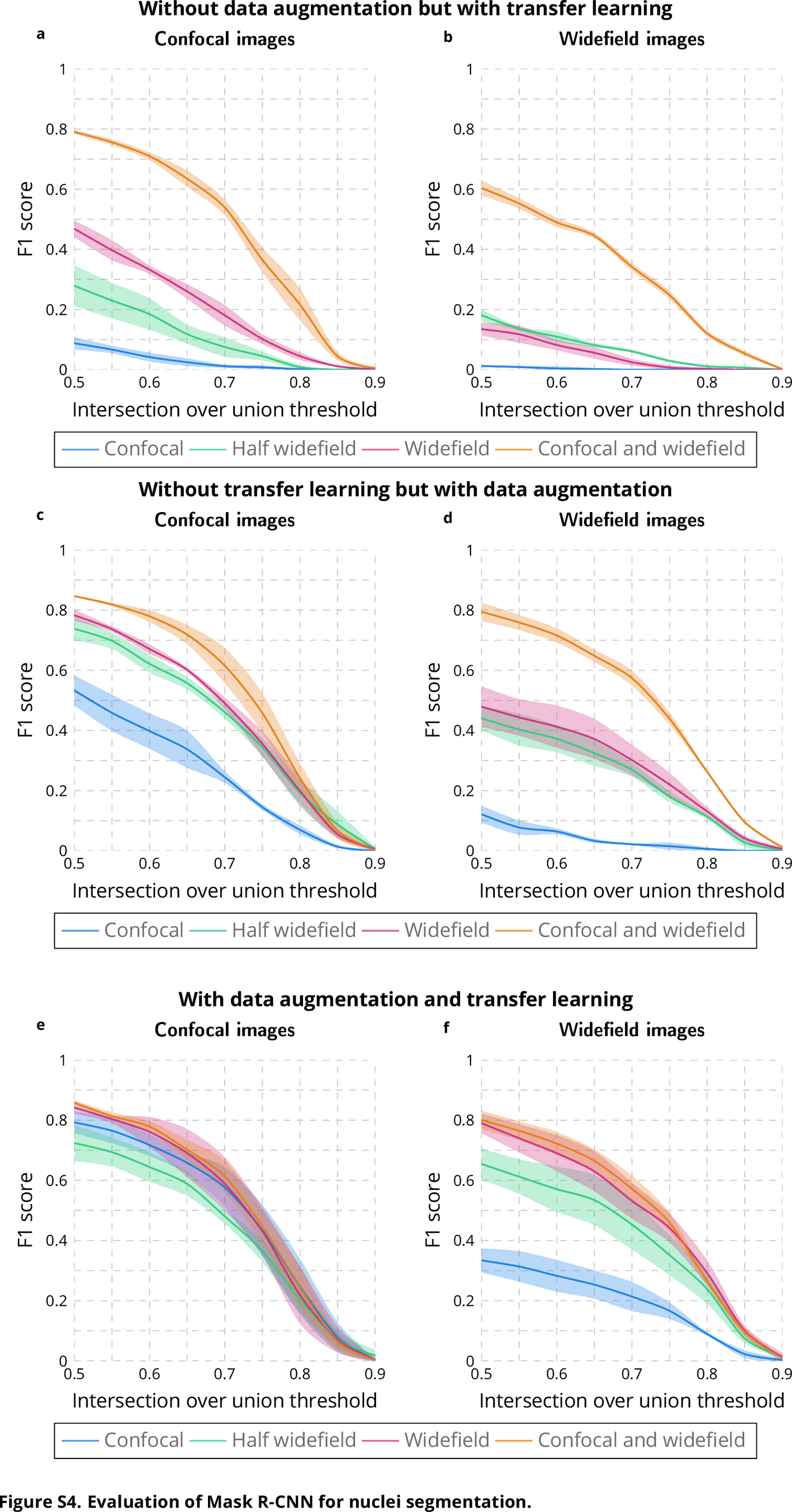

Supplement: S4 Fig — F1 score for multiple IoU thresholds obtained with the Mask R-CNN approach by trainig on confocal, half or all widefield images, and on confocal and widefield images without data augmentation but with transfer learning a-b, without transfer learning but with data augmentation c-d, with data augmentation and transfer learning e-f. The lines correspond to the average F1 score while the areas represent the standard error. (TIF) [file pcbi.1009949.s004.tif]

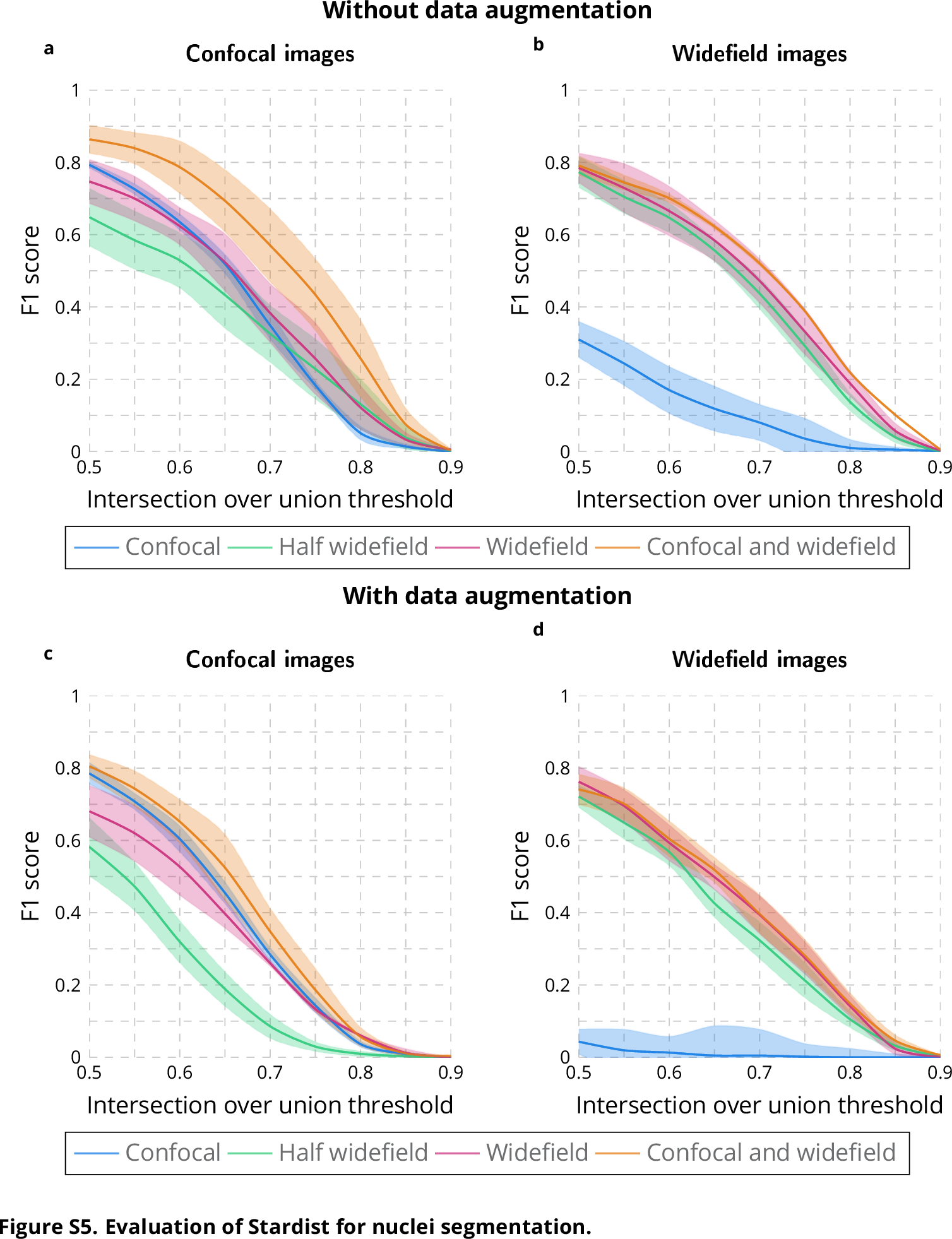

Supplement: S5 Fig — F1 score for multiple IoU thresholds obtained with the Stardist approach by trainig on confocal, half or all widefield images, and on confocal and widefield images without data augmentation a-b and with data augmentation c-d. The lines correspond to the average F1 score while the areas represent the standard error. (TIF) [file pcbi.1009949.s005.tif]

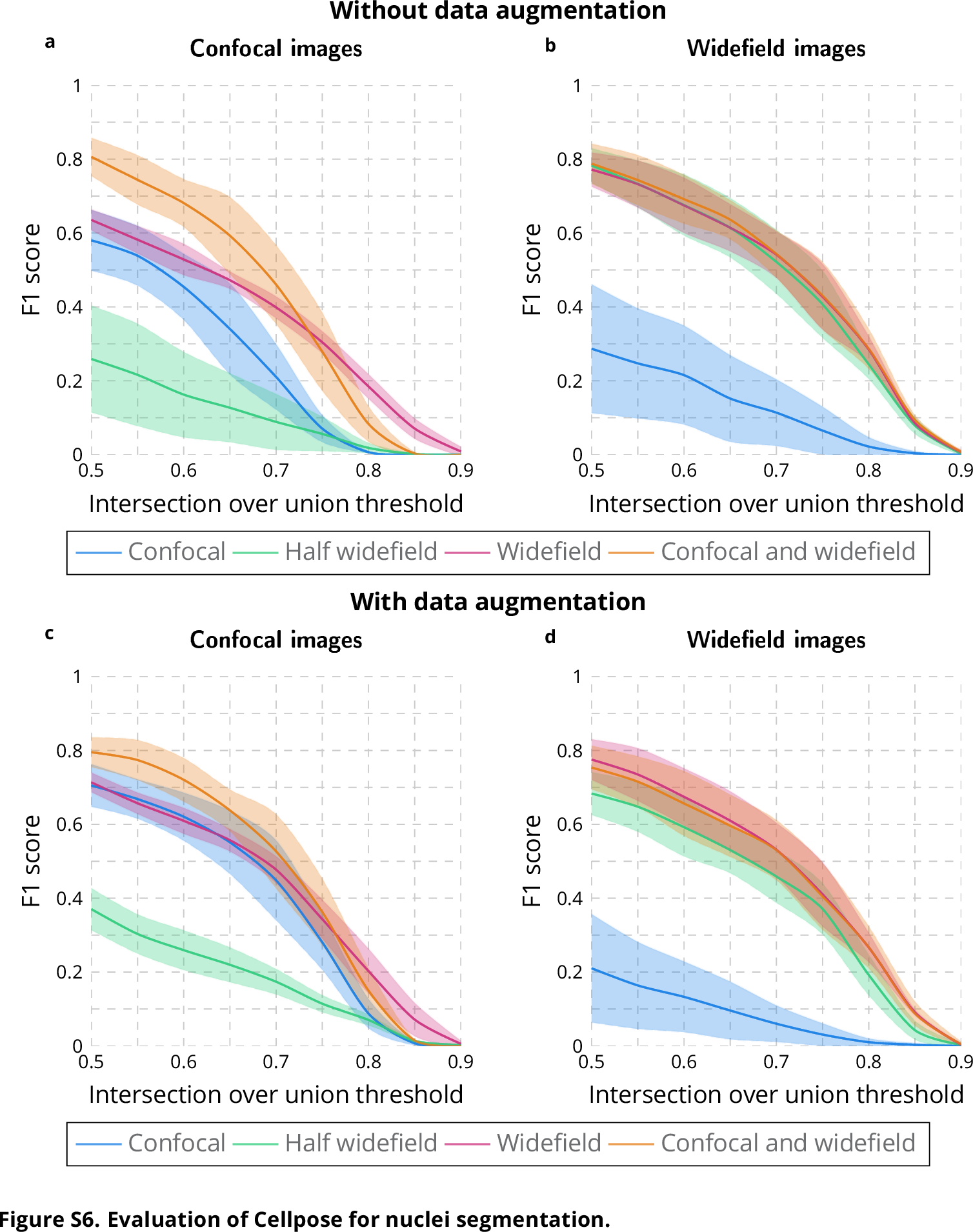

Supplement: S6 Fig — F1 score for multiple IoU thresholds obtained with the Cellpose approach by trainig on confocal, half or all widefield images, and on confocal and widefield images without data augmentation a-b and with data augmentation c-d. The lines correspond to the average F1 score while the areas represent the standard error. (TIF) [file pcbi.1009949.s006.tif]

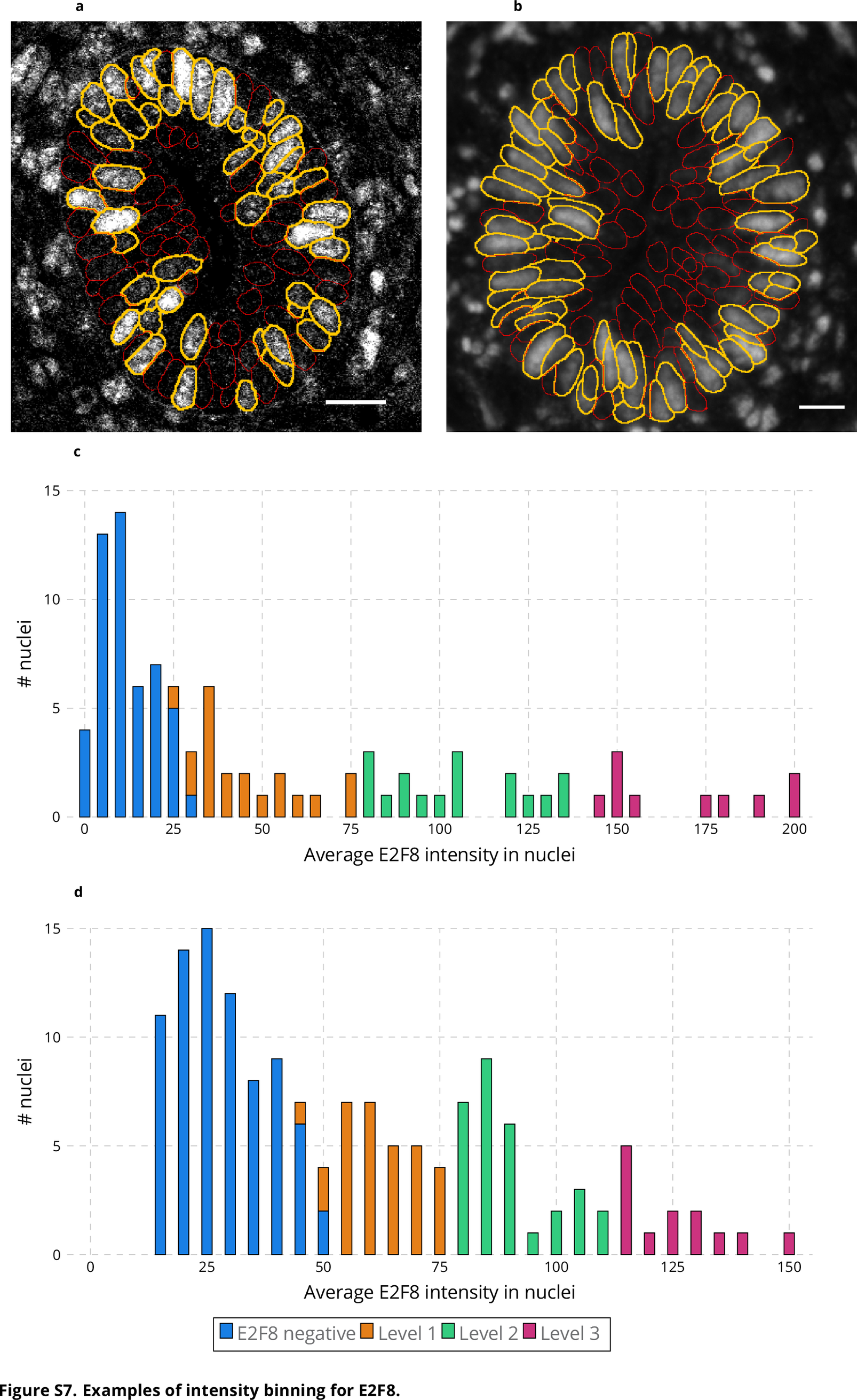

Supplement: S7 Fig — a-b E2F8 channel of a confocal a and a widefield b image. E2F8 positive nuclei are overlaid as orange circles while E2F8 negative nuclei are overlaid as red circles. Scale bar = 20μm. c-d Histograms of the E2F8 intensity observed in the nuclei shown in images a (c) and b (d). The 3 levels of intensity used for concentration estimation are displayed with different colors. (TIF) [file pcbi.1009949.s007.tif]

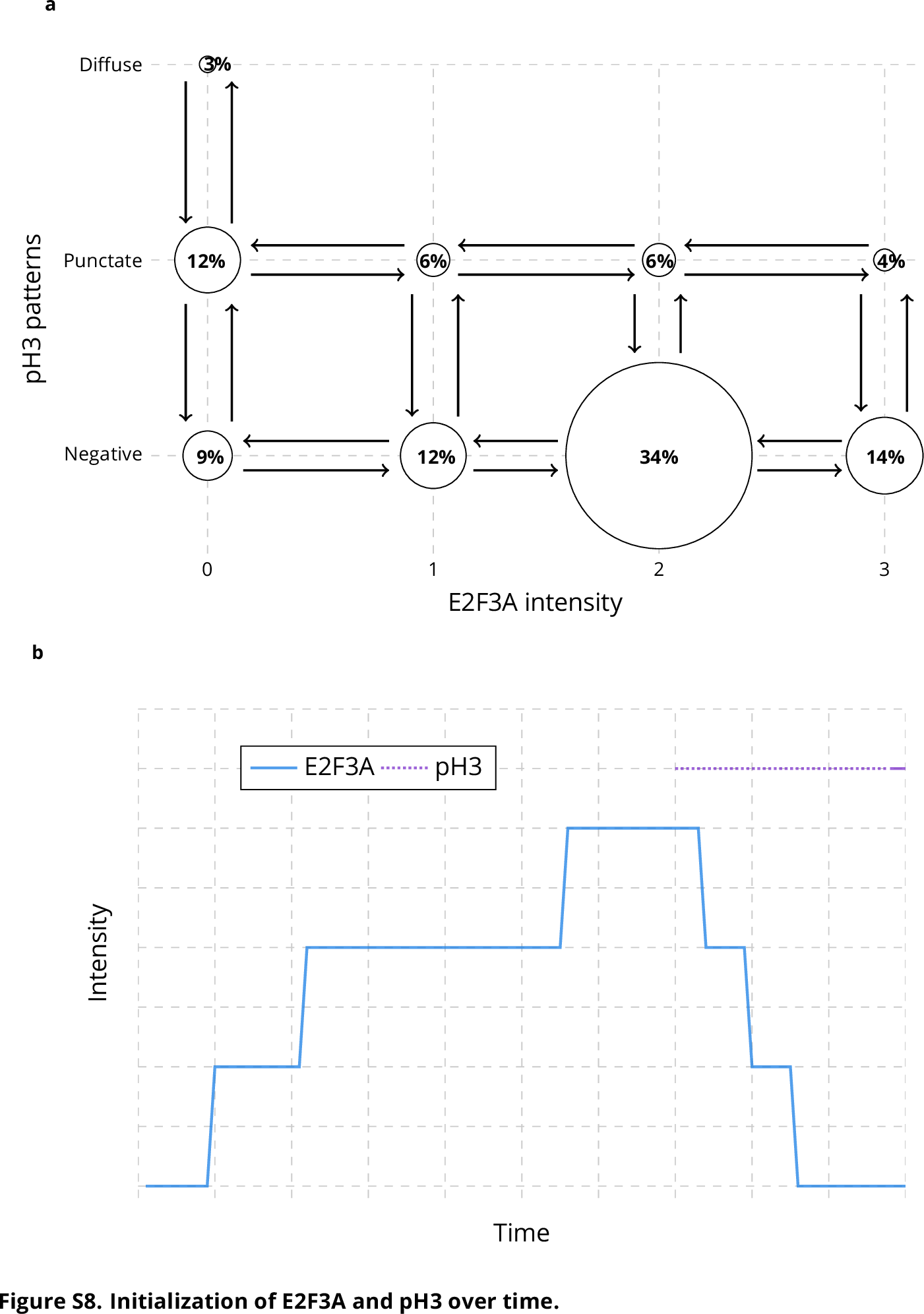

Supplement: S8 Fig — a 2D histogram of E2F3A intensity and pH3 patterns. b Initialization of E2F3A and pH3 over time from the 2D histogram shown in a. (TIF) [file pcbi.1009949.s008.tif]

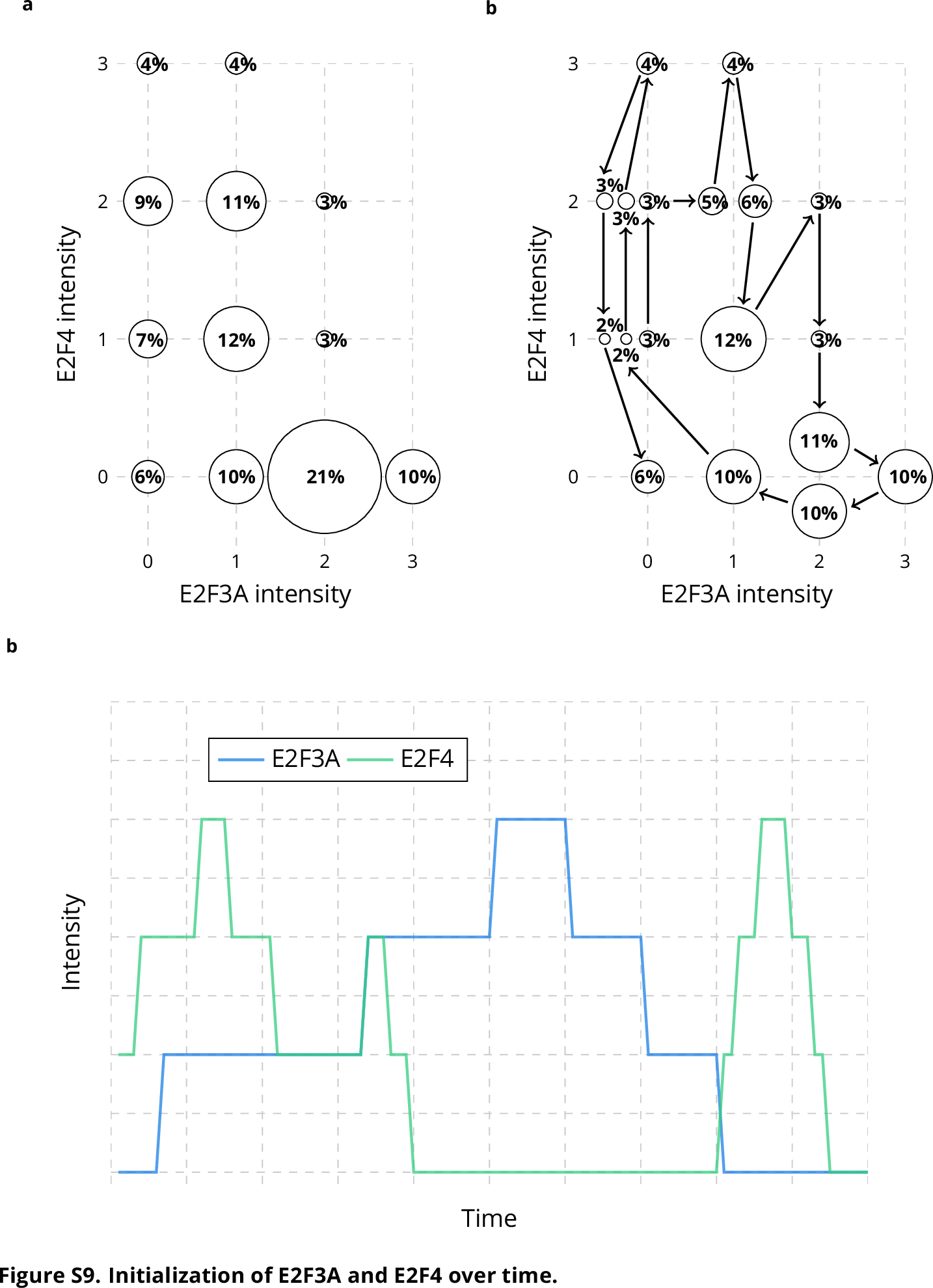

Supplement: S9 Fig — a 2D histogram of E2F3A and E2F4 intensity. b Modified 2D histogram of E2F3A and E2F4 intensity with vertex splitting and set of edges giving the minimum cost. c Initialization of E2F3A and E2F4 over time from the set of edges shown in b. (TIF) [file pcbi.1009949.s009.tif]

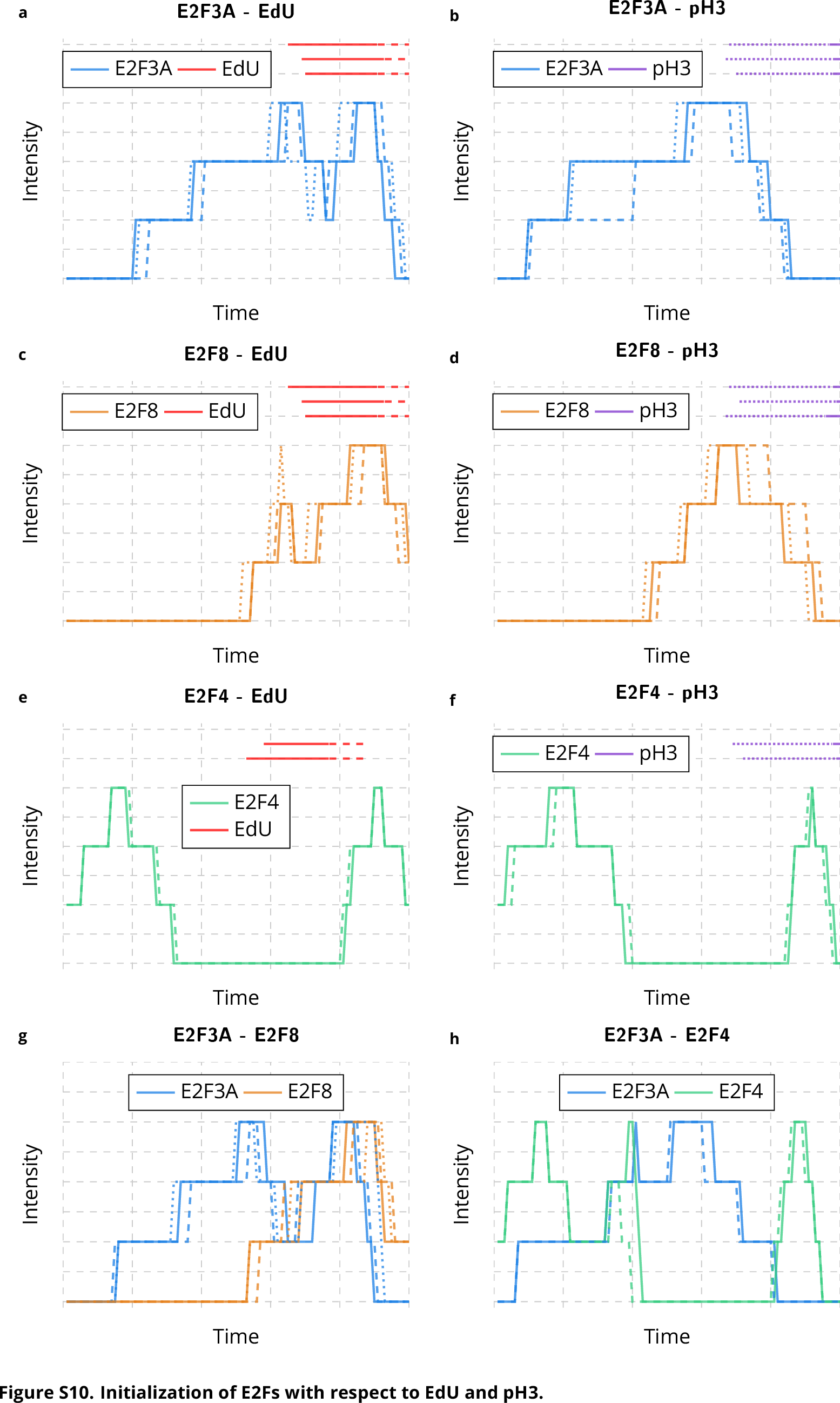

Supplement: S10 Fig — a-b Initialization of E2F3A with respect to a EdU (n = 3) and b pH3 (n = 3). c-d Initialization of E2F8 concentration with respect to c EdU (n = 3) and d pH3 (n = 3). e-f Initialization of E2F4 concentration with respect to e EdU (n = 2) and f pH3 (n = 2). g-h Initialization of E2F3A concentration with respect to g E2F8 (n = 3) and h E2F4 (n = 2). For all curves, the E2Fs intensity for the different mice are represented as curves with different line styles while EdU and pH3 are shown above the E2Fs curves, with solid lines corresponding to diffuse states and dashed lines corresponding to punctate states. (TIF) [file pcbi.1009949.s010.tif]

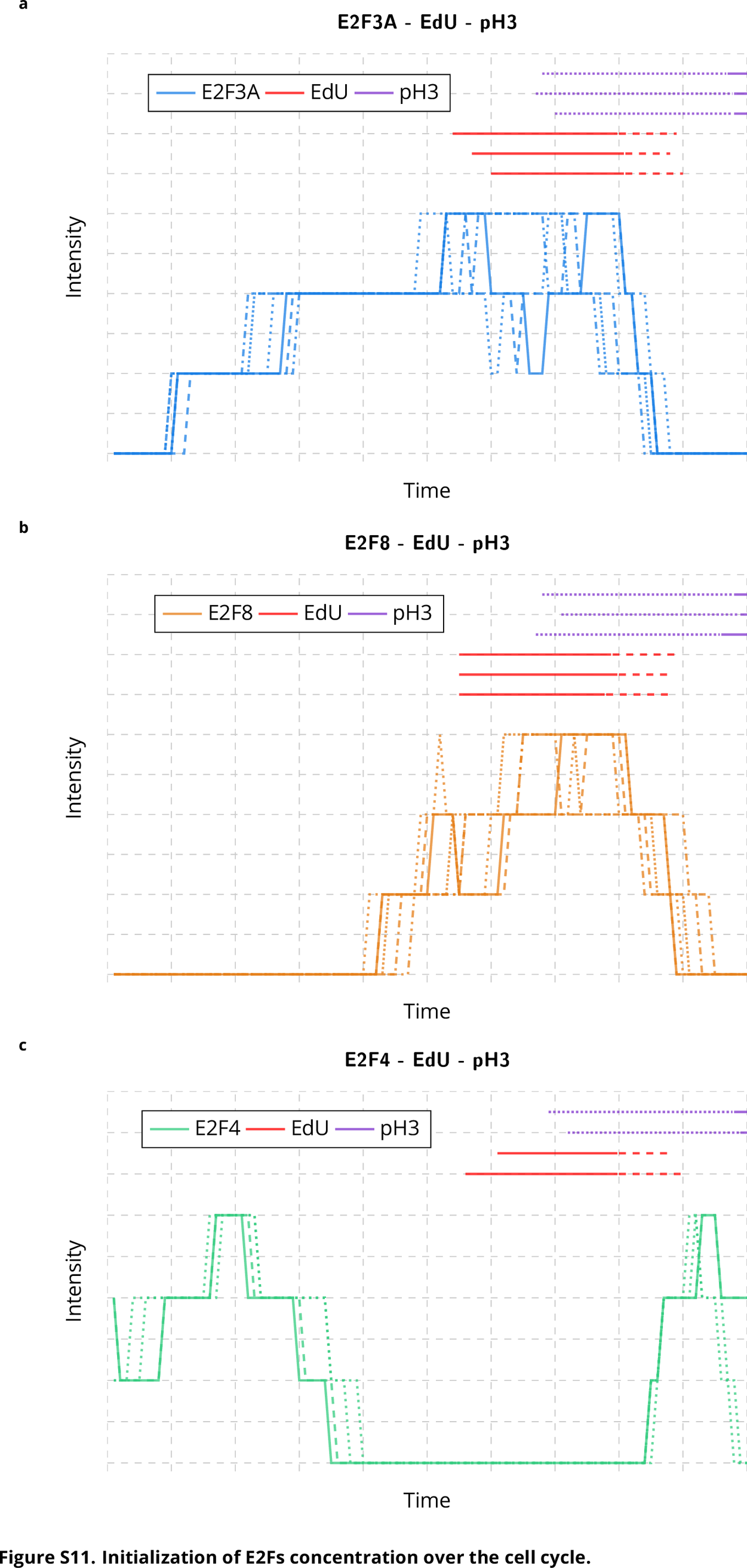

Supplement: S11 Fig — a Temporal evolution of E2F3A over the cell cycle after registration between EdU and pH3 (n = 6). b Temporal evolution of E2F8 over the cell cycle after registration between EdU and pH3 (n = 6). c Temporal evolution of E2F4 over the cell cycle after registration between EdU and pH3 (n = 4). For all curves, the E2Fs intensity for the different mice are represented as curves with different line styles while EdU and pH3 are shown above the E2Fs curves, with solid lines corresponding to diffuse states and dashed lines corresponding to punctate states. (TIF) [file pcbi.1009949.s011.tif]

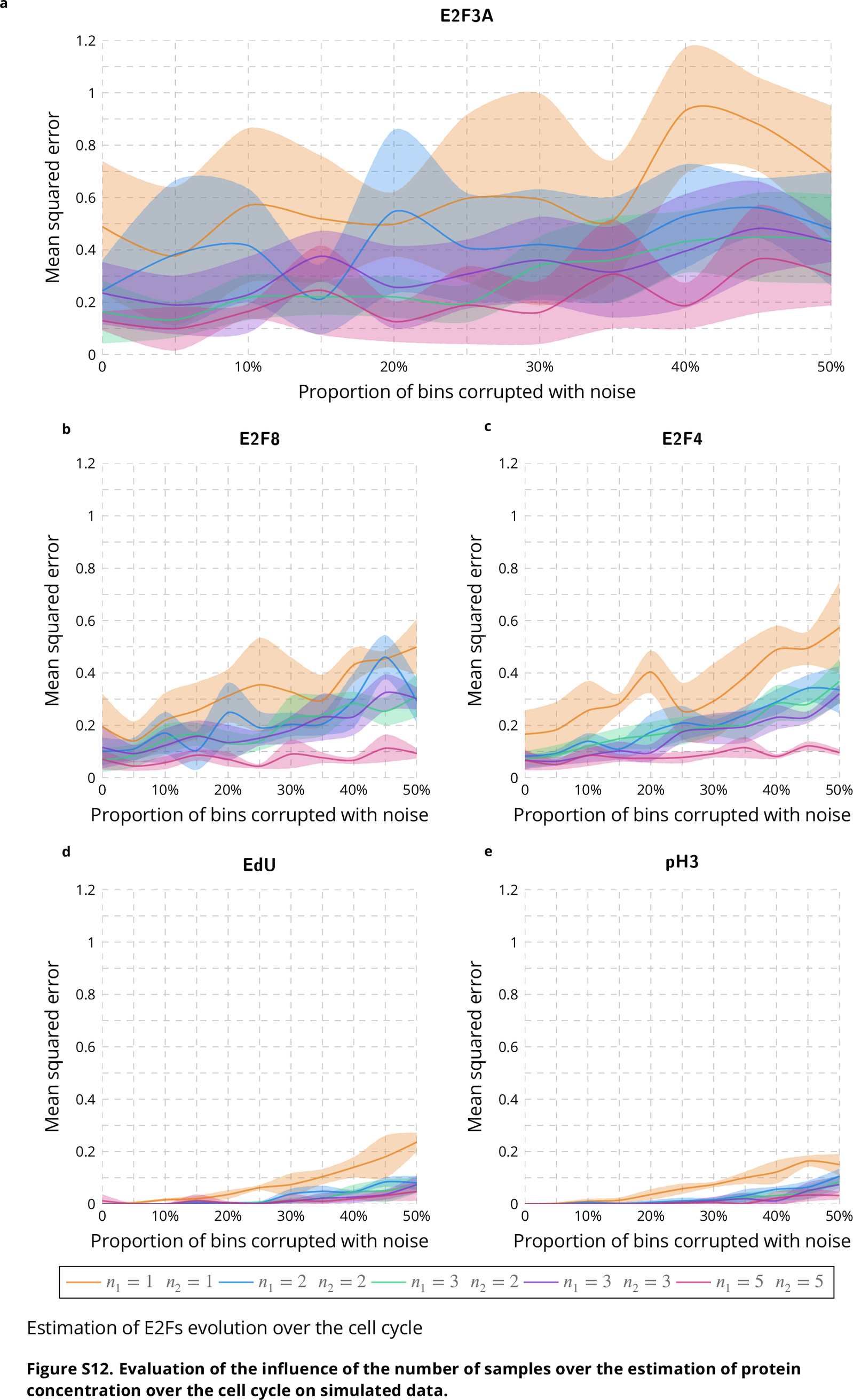

Supplement: S12 Fig — Mean squared error between the estimated and simulated concentrations of E2F3A a, E2F8 b, E2F4 c, EdU d and pH3 e by considering different numbers of samples when corrupting up to 50% of the simulated data with noise. n1 corresponds to the number of samples for E2F3A, E2F8, EdU and pH3 while n2 is the number of samples for E2F4. The lines correspond to the average mean squared error while the areas represent the standard error. (TIF) [file pcbi.1009949.s012.tif]

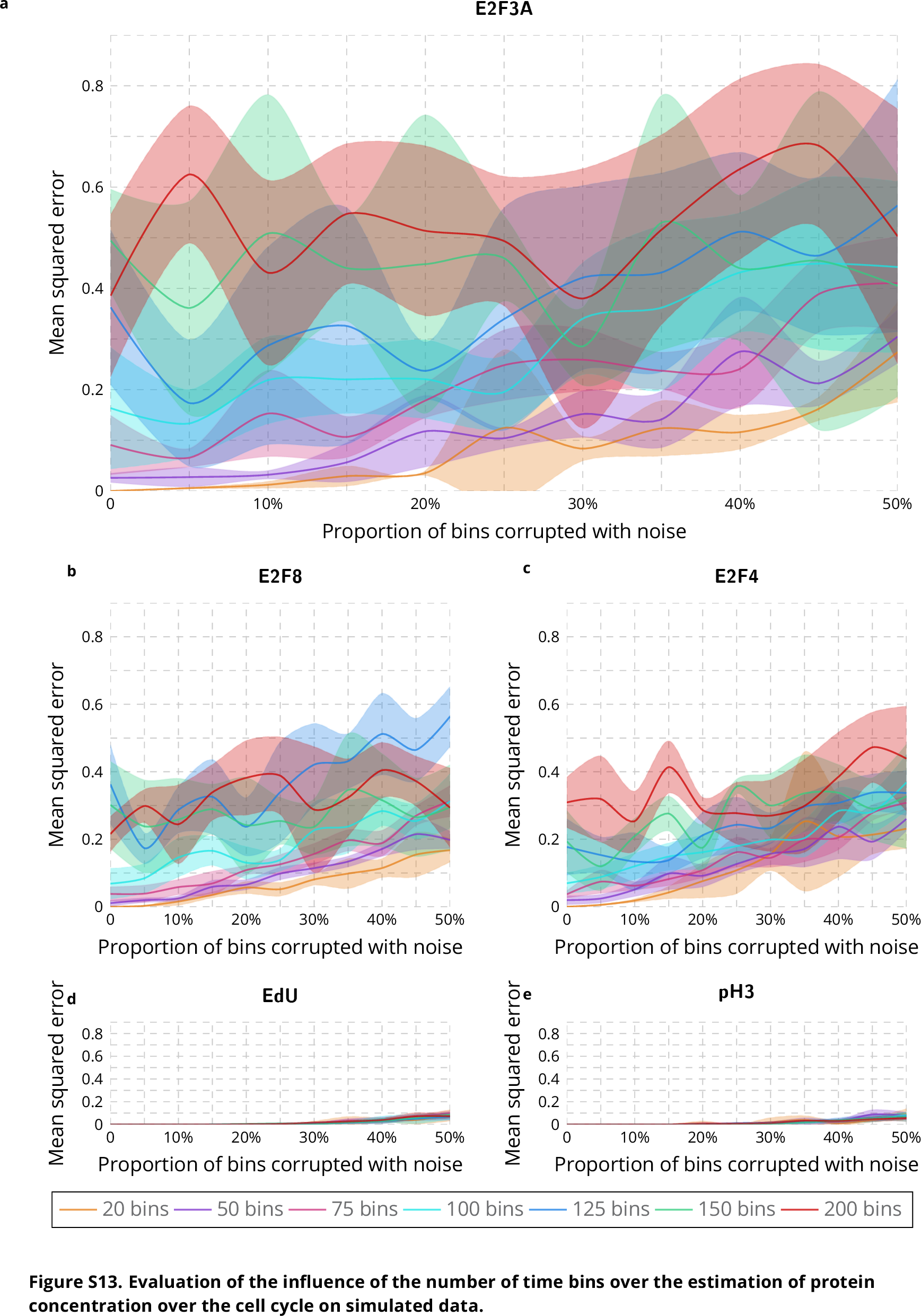

Supplement: S13 Fig — Mean squared error between the estimated and simulated concentrations of E2F3A a, E2F8 b, E2F4 c, EdU d and pH3 e by considering different numbers of time bins when corrupting up to 50% of the simulated data with noise. The lines correspond to the average mean squared error while the areas represent the standard error. (TIF) [file pcbi.1009949.s013.tif]

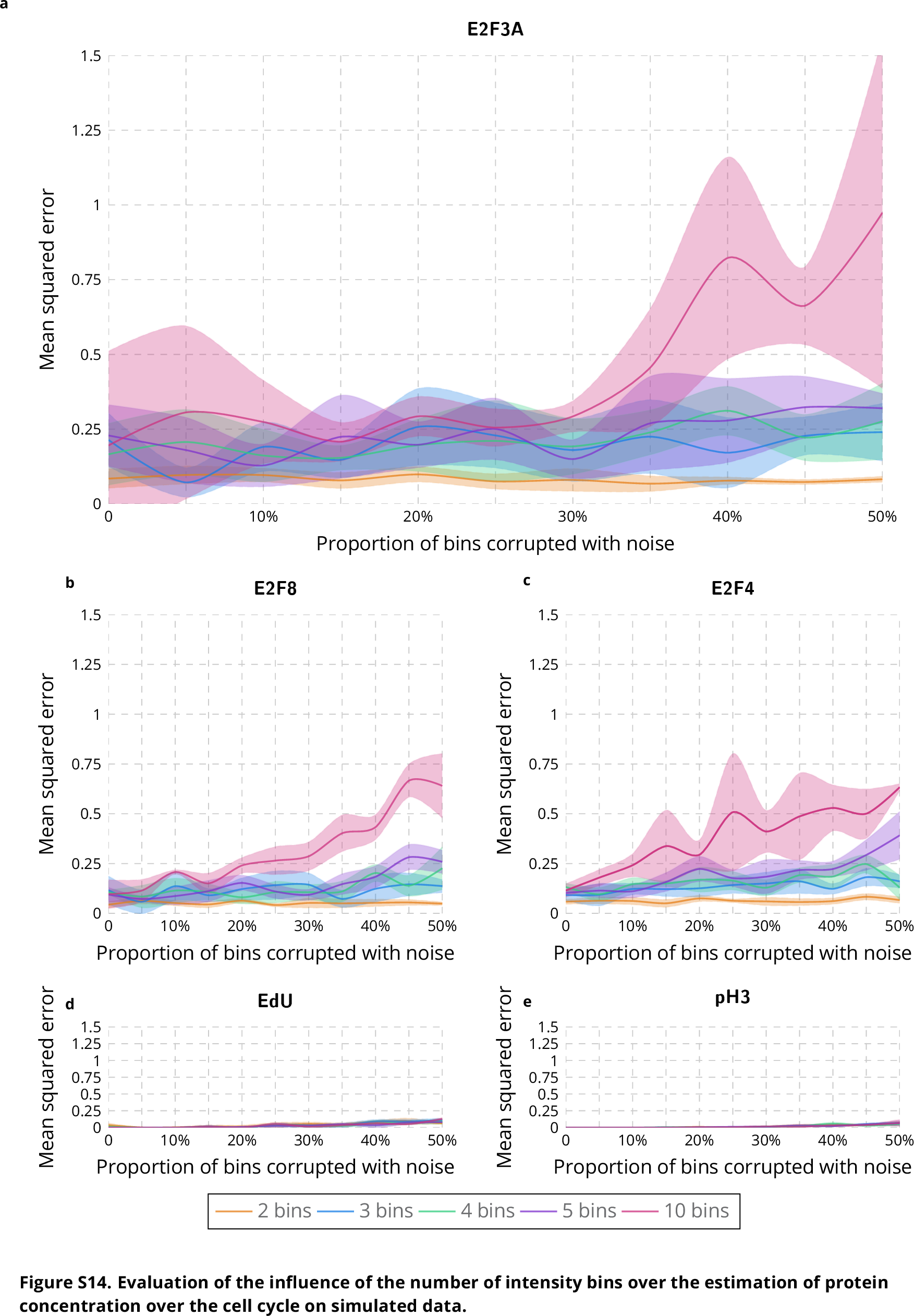

Supplement: S14 Fig — Mean squared error between the estimated and simulated concentrations of E2F3A a, E2F8 b, E2F4 c, EdU d and pH3 e by considering different numbers of intensity bins when corrupting up to 50% of the simulated data with noise. The lines correspond to the average mean squared error while the areas represent the standard error. (TIF) [file pcbi.1009949.s014.tif]

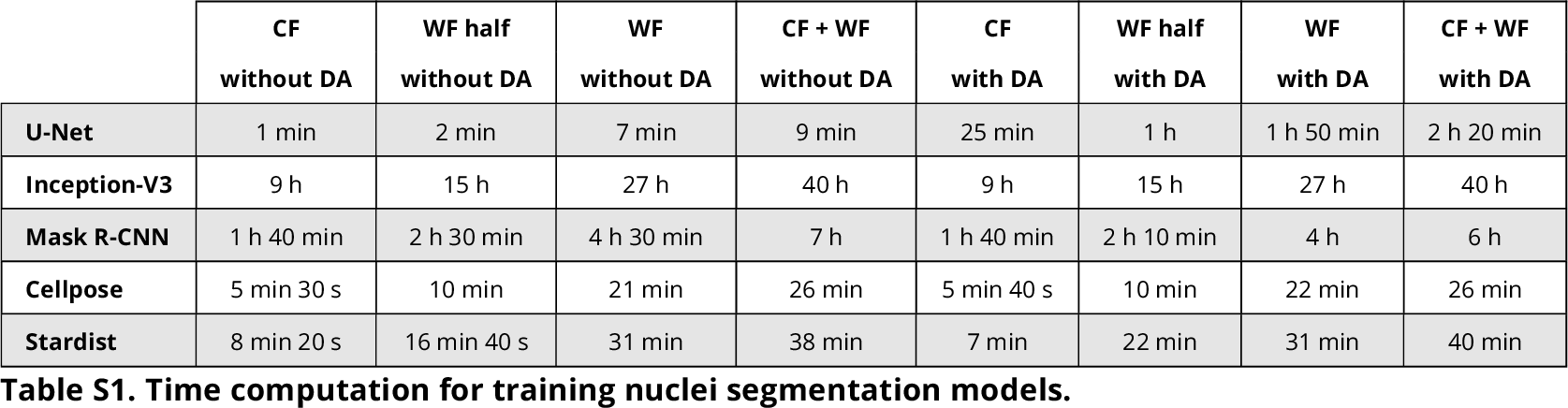

Supplement: S1 Table — Computation time needed to train the five different deep learning approaches on the four training datasets with a GeForce RTX 2080 with Max-Q design. CF stands for confocal images, WF half stands for half the widefield images, WF stands for widefield images and CFWF stands for confocal and widefield images. (TIF) [file pcbi.1009949.s015.tif]

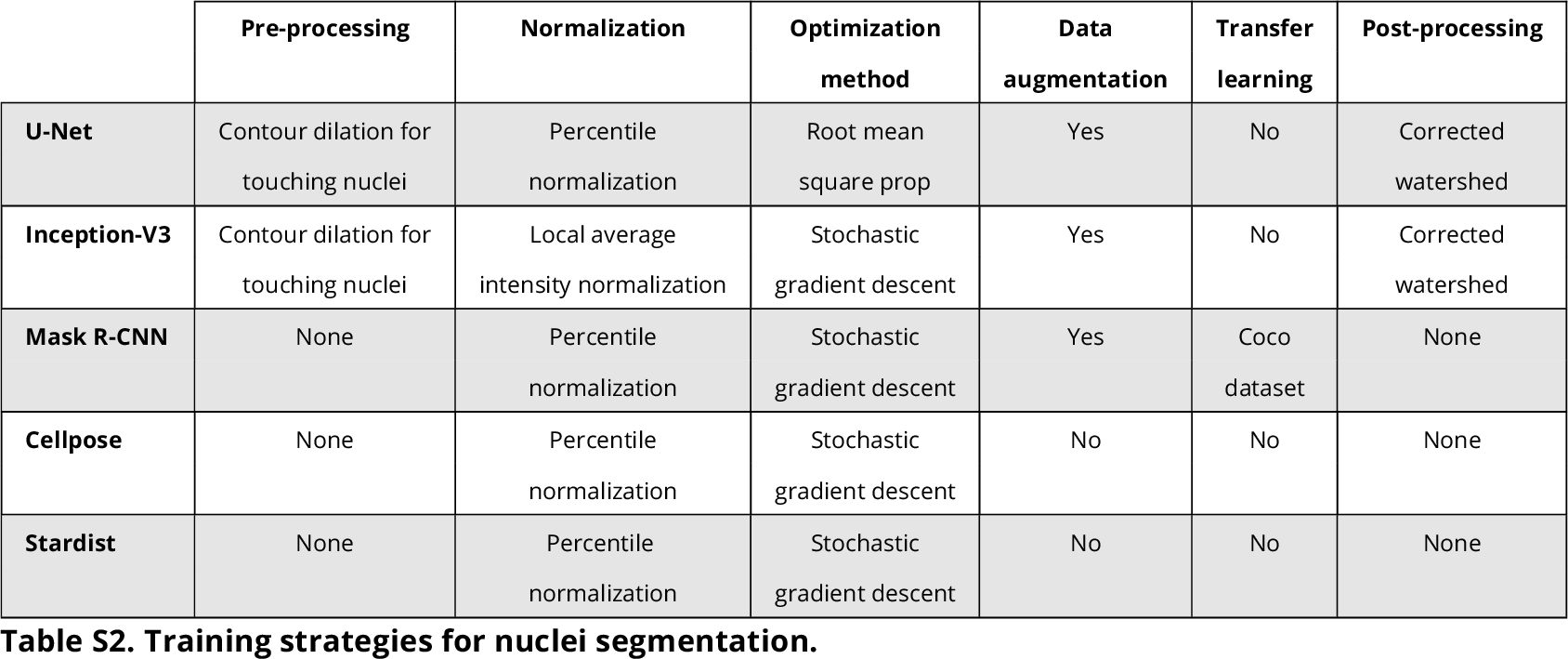

Supplement: S2 Table — Pre-processing, normalization, optimization method, data augmentation, transfer learning and post-processing used to train the five deep learning approaches. (TIF) [file pcbi.1009949.s016.tif]

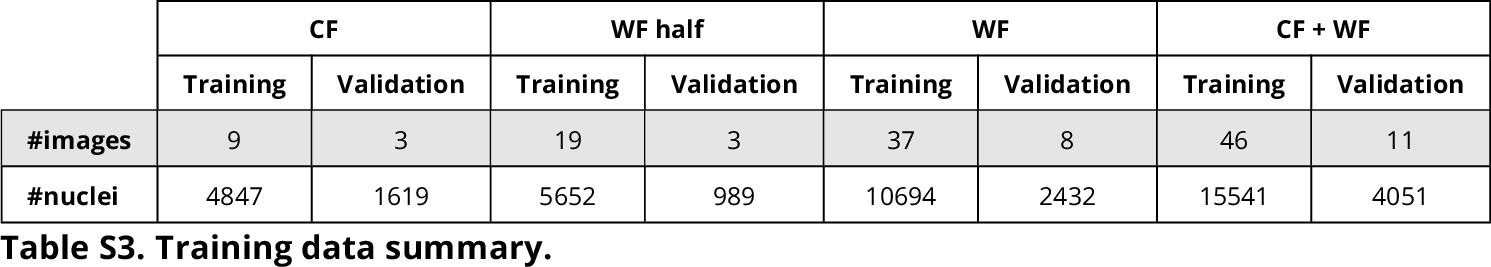

Supplement: S3 Table — Number of images and nuclei in the four different training and validation datasets. CF stands for confocal images, WF half stands for half the widefield images, WF stands for widefield images and CFWF stands for confocal and widefield images. (TIF) [file pcbi.1009949.s017.tif]

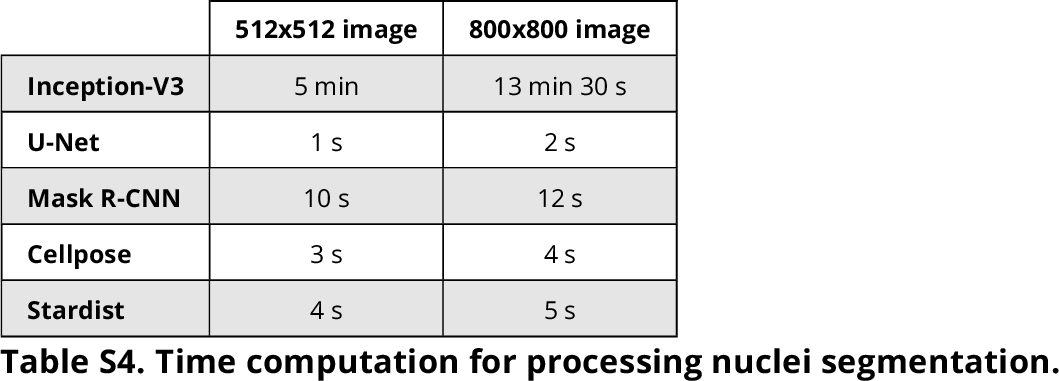

Supplement: S4 Table — Computation time needed to process the five different deep learning approaches on the four training datasets with a GeForce RTX 2080 with Max-Q design. (TIF) [file pcbi.1009949.s018.tif]

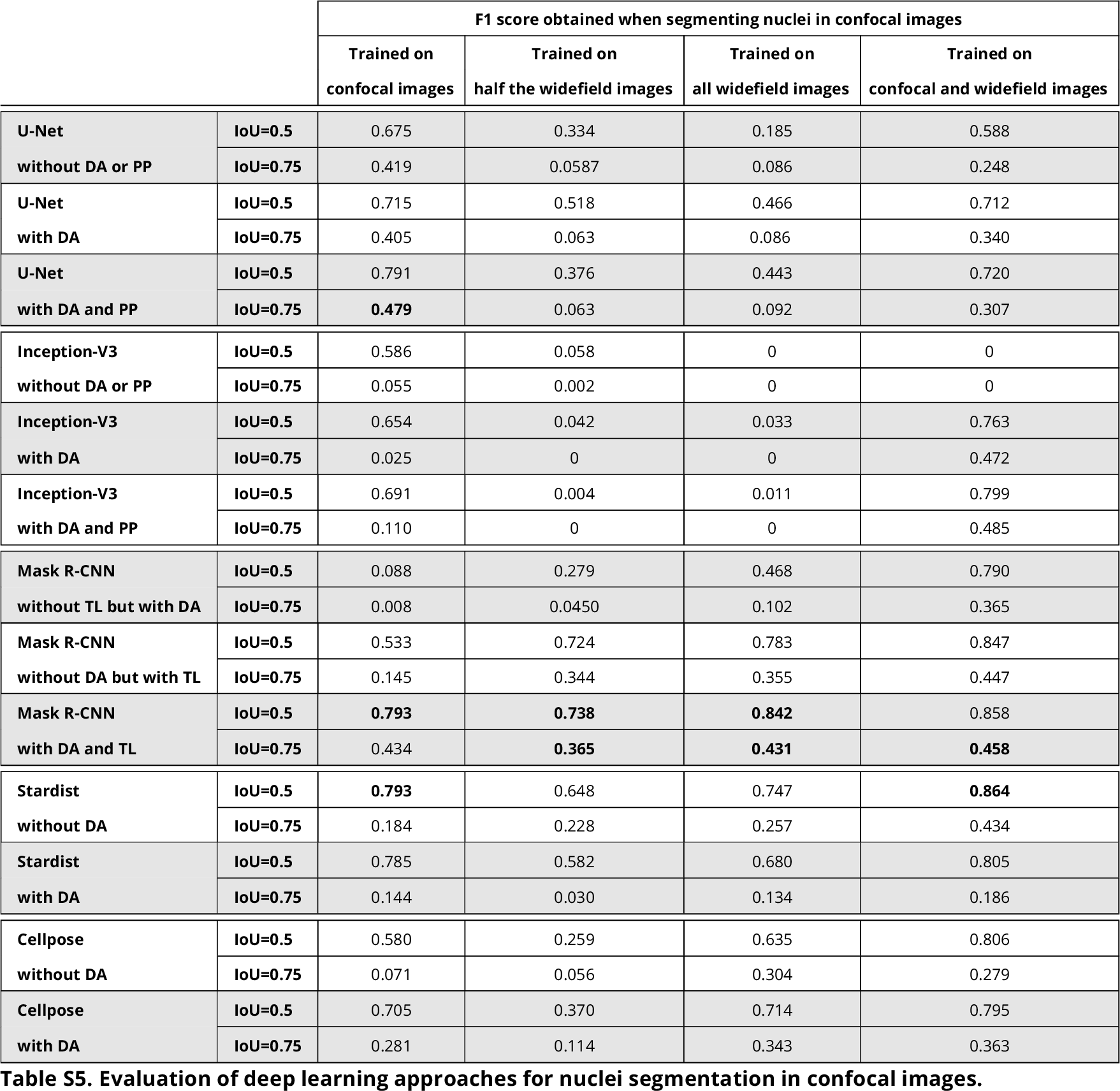

Supplement: S5 Table — F1 score obtained for IoU = 0.5 and IoU = 0.75 when segmenting nuclei in confocal images with U-Net, Inception-V3, Mask R-CNN, Stardist and Cellpose. DA stands for data augmentation, PP stands for post-processing, TL stands for transfer learning and IoU stands for intersection over union. (TIF) [file pcbi.1009949.s019.tif]

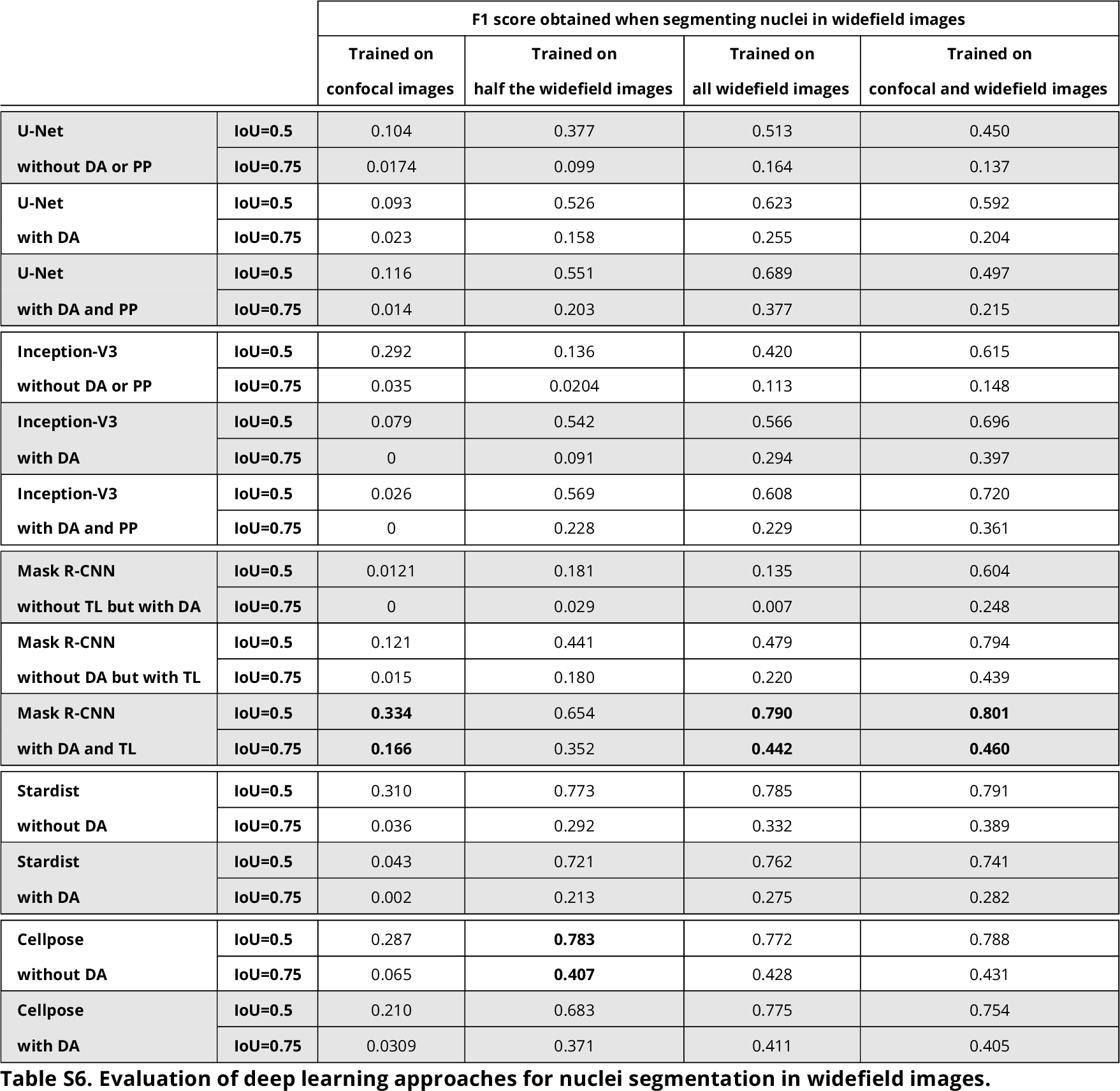

Supplement: S6 Table — F1 score obtained for IoU = 0.5 and IoU = 0.75 when segmenting nuclei in widefield images with U-Net, Inception-V3, Mask R-CNN, Stardist and Cellpose. DA stands for data augmentation, PP stands for post-processing, TL stands for transfer learning and IoU stands for intersection over union. (TIF) [file pcbi.1009949.s020.tif]

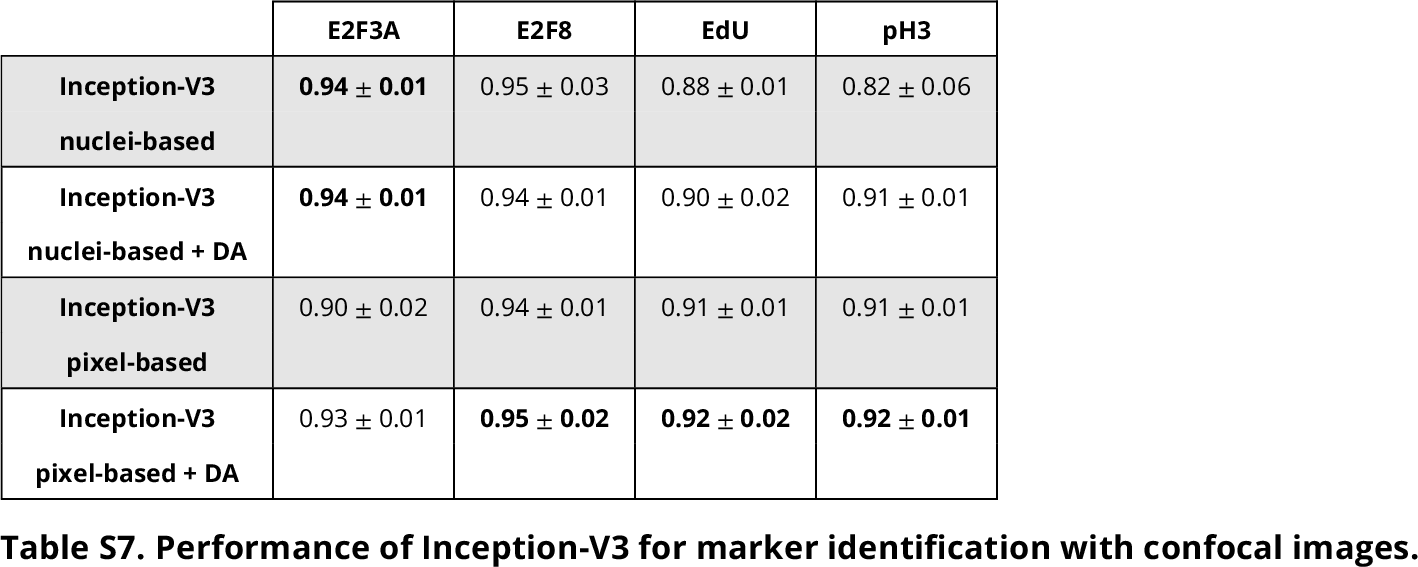

Supplement: S7 Table — Inception-V3 accuracy and standard error with and without transfer learning for marker identification of E2F3A, E2F8, EdU and pH3 in confocal images. DA stands for data augmentation. (TIF) [file pcbi.1009949.s021.tif]

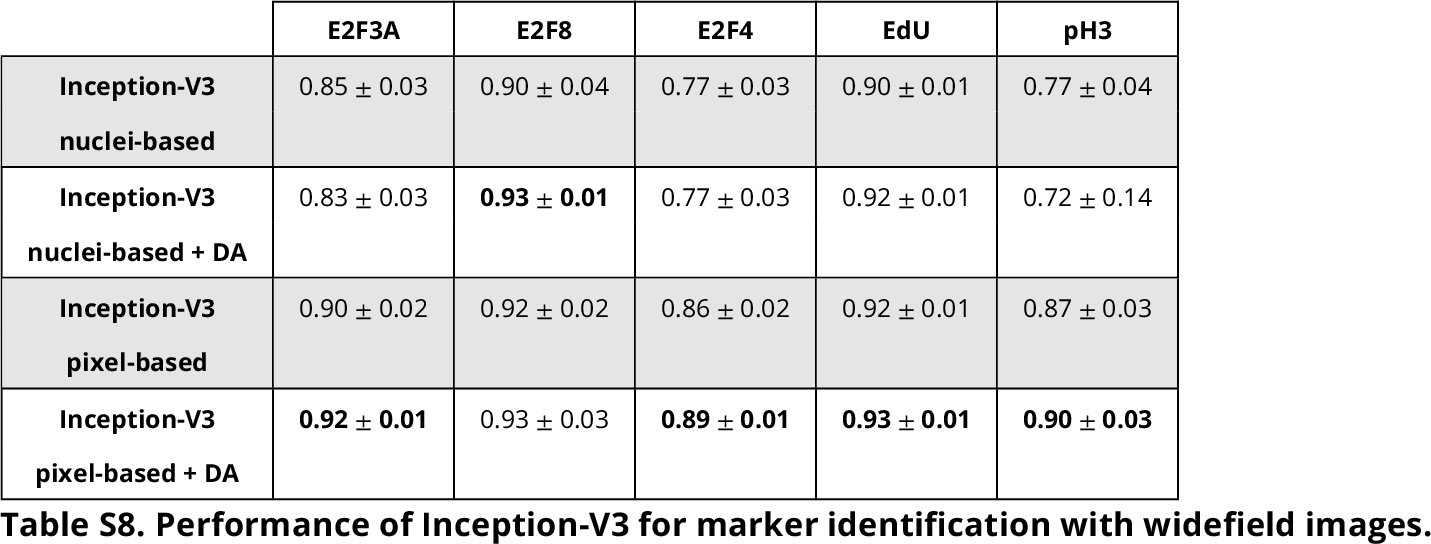

Supplement: S8 Table — Inception-V3 accuracy and standard error with and without transfer learning for marker identification of E2F3A, E2F8, E2F4, EdU and pH3 in widefield images. DA stands for data augmentation. (TIF) [file pcbi.1009949.s022.tif]

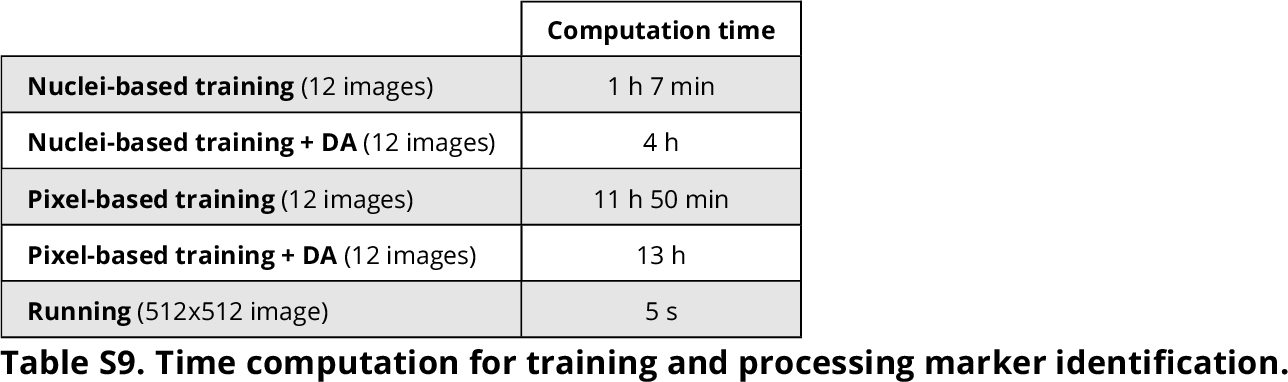

Supplement: S9 Table — Computation time needed to train and process Inception-V3 for marker identification with a GeForce RTX 2080 with Max-Q design. DA stands for data augmentation. (TIF) [file pcbi.1009949.s023.tif]
