# Supplementary material for: Deep learning tools and modeling to estimate the temporal expression of cell cycle proteins from 2D still images
Source: PLoS Comput Biol. 2022 Mar 14;18(3):e1009949. doi: 10.1371/journal.pcbi.1009949 (PMC8947602; doi:10.1371/journal.pcbi.1009949)
Supplement: Attachment — Submitted filename: AnswersToReviewers.pdf [file pcbi.1009949.s024.pdf]

Reviewer #1: The authors have three main contributions.

1- Benchmarking three existing computer vision architectures for cell segmentation (U-Net, Mask-R-CNN, Inception V3)

2- Benchmarking a computer vision architectures for estimating protein concentration (Inception V3)

3- Software tools that implement their pipeline (github repo + imagej plugin).

4- An optimization based approach to estimate protein concentration over time.

In part 1 and 2, the results are presented in an extremely confusing way. For example:
- At the very least, the authors should have a table, showing most of the relevant information in a single place. This would include: number of cells (not just images!) used for training, training time, prediction, etc. Figure 2 includes 2 subplots to show 16 numbers.

We acknowledge that the results were not presented in the most straightforward way and thank reviewer #1 for this valuable advice. First, we added evaluations for better evaluating the performance of the 5 deep learning approaches used for nuclei segmentation (Figures S2-S6). We also added Table S3 to indicate the number of images as well as the number of nuclei in the different training and validation datasets. Tables S1;S4 show the computation time for training and processing the 5 deep learning methods evaluated for nuclei segmentation. Box plots in Fig.2 were replaced with tables. Figure S5 in the initial submission was replaced with Tables S5-S6 for evaluating the performance of Inception-V3 for marker identification and with Table S7 for time computation.

- It would be nice to see training curves for the different methods. The authors speculate that certain methods benefit more from data, but it would be nice to see this evaluated.

We feel that the number of supplementary figures is already large and that adding training curves would not bring much additional value. However, these curves could be added if reviewer(s) ask to do so.

-Computer vision is a very quickly moving field, and I don't expect people in other fields to use and benchmark all the latest methods - but - it would be nice to see more methods get benchmarked. Ross Wightman has all the major methods implemented here with a consistent API and pretrained weights: https://github.com/rwightman/pytorch-image-models

We chose to keep things simple and compare classification (Inception-V3), semantic segmentation (U-Net) and instance segmentation (Mask R-CNN) for nuclei segmentation. However, as also suggested by Reviewer #3, we added two popular methods in microscopy: Stardist and Cellpose. It is not a surprise that instance segmentation approaches lead to a better accuracy. Consequently, it makes sense to show a comparison between instance segmentation approaches while having only one method for classification and instance segmentation as baselines.

- The methods are not described in nearly enough detail, and the text is difficult to follow. We should have clear diagrams that show the training pipeline for each method, which method used pre-training, which didn't, what

method used post processing, etc - some of this information is scattered throughout the text, but it's a huge pain to have to go back and forth.

We thank Reviewer #1 for this important point. We added Table S2 to show in one place the pre-processing, normalization, optimization method, use of data augmentation, use of transfer learning and post-processing for each deep learning method used for nuclei segmentation.

- The Python code (as presented in the repo) is not really packaged in a way that someone else could use it for their own use case. I appreciate the authors making the code available - but - in its current form, it's not really a resource for anyone else. At the very least, there should be a better Readme. Ideally, the code would be packaged in a library with a unified API.

We changed the code in the repo so that it can be used by biologists that do not have background in coding. Mainly, we use widgets so parameters can be defined by directly clicking or entering values. We also added video tutorials with links in the Readme so that users can learn how to install cuda, Python and the required Python packages and see how to use the different notebooks to train models and process them.

- The authors discuss quantizing E2F signal in 4 buckets, but never show any kind of data justifying this decision - at the very least a histogram of concentration should be shown.

Scoring of immuno-staining according to intensity into four basic categories or bins, namely negative, weak, positive and strong (or 0-3+ binning), is widely used in both diagnostic pathology and biomedical research as it provides an estimate of protein expression that is biologically relevant and sufficient in most situations. Considering that current immunostaining techniques have limitations in terms of dynamic range, a rather simple binning such 0-3+ is preferable. Even though binning using image analysis can be more extensive (e.g. ten-bin), one has to wonder to what extent such small variations in intensity of immunostaining truly reflect changes in protein expression. Previous research by our group and others [36,37] has described the cell cycle kinetics of E2F protein expression using *in vitro* systems. Based on this prior knowledge, binning E2F expression levels into high, moderate or low provided us with enough information to reconstruct the patterns of expression of these proteins in tissues *in situ*.

- The last part of the paper showing how the graph assignment is solved is clever, and I appreciated the author explaining why they felt justified in making certain assumptions. I wish they slightly more precise - for example - what exactly does "2 Temporal evolution of protein accumulation is similar in all observed cells." mean?

We changed the text in the third section to better explain our approach designed to estimate the temporal concentration of proteins over the cell cycle. We also added an extensive evaluation with simulated data. Mainly, we assessed the impact of the number of samples, the number of intensity and time bins in the presence of noisy data. Finally, we added a new section to test if the temporal evolution of protein concentration can be estimated without marker identification, which also allowed us to evaluate the influence of the training dataset used for nuclei segmentation with respect to the temporal evolution of protein concentration over the cell cycle.

Overall, I feel the technical issues in the paper + the lack of clarity means the paper should not be accepted in its current form.

We think that the additional figures and tables drastically help to better understand the differences between the different deep learning approaches used for nuclei segmentation. We feel that adding simulated data to evaluate the modelling part and the study of the impact of the training datasets used for nuclei segmentation on the estimation of protein concentration over the cell cycle 1) emphasize the interest of this approach; 2) justify putting together in a same manuscript the preprocessing steps, *i.e.* nuclei segmentation and marker identification, and the modelling part.

Reviewer #2: The manuscript focuses on the temporal expression of E2F proteins during the cell cycle. In a first part, the segmentation of nuclei is performed based on a deep-learning approach. The performance of several networks are compared, and the Mask R-CNN is chosen as more appropriate. In a second part, the fluorescence pattern of proteins is investigated, using a combination of deep-learning approach and manual correction of the results. A software was developed to help correct and validate the results obtained by automated segmentation. Finally, the temporal evolution of protein concentration is investigated by considering a large population of cells at various stages in a static image, and reconstructing the time variations of fluorescence between stages using graph optimization methods.

The manuscript presents a number of results together with an exhaustive explanation on the methods. However, I found it difficult to read, making it complicated to clearly identify what were the questions addressed within the text. For instance, it is not clear if the paper aims at solving a biological question by using computational methods, or if it focuses on the methodological part, in particular deep-learning segmentation, using the biological question as an illustration. This confusion deserves the presentation of the results, that however seem to present interest. If the biology is the main concern, I suggest putting more emphasis on it in the introduction, and introduce the methodological questions based on it. One possibility could be to start from the limitations encountered in ref [11], and use them to introduce the proposed enhancements. Another option could be to focus on the methodological questions. Actually the results put emphasis on the performance and accuracy of the methods. However, this option would imply splitting the manuscript into two distinct papers.

This paper focuses on the methodological part and Reviewer #2 makes a fair point when suggesting to split the manuscript into two distinct papers: one about the nuclei segmentation and marker identification, one about the estimation of the protein concentration over the cell cycle. However, we believe it is important to present the workflow in its entirety to assess the validity of the approach as nuclei segmentation and marker identification are impacting the final result in this study. In order to evaluate this impact, we added a new section in which we propose to estimate the protein concentration evolution over the cell cycle without marker identification, so we can assess the influence of the training datasets used for nuclei segmentation on the final estimation.

Other comments

** the introduction reviews segmentation of fluorescent structures approaches by considering deep-learning approaches. While very popular, DL-approach is not the only approach for segmentation, and discussing with respect to other "classical" approaches could help relate to the field.*

We added a sentence l.21-23 to mention the watershed approach as a traditional way to segment nuclei.

** L50-52: well, I guess everyone would expect a deep-learning approach to perform better than a threshold approach…*

We acknowledge that this result is expected, we only want to demonstrate it on our data.

** L56: it would be more clear to explain the three assumptions directly in the introduction.*

We agree that this could help to directly get the main idea about our approach, but we feel that the introduction would then be unbalanced by having a large focus on the estimation of protein concentration over

the cell cycle. However, it is the main contribution of the paper and we would not mind adding the three assumptions in the introduction if reviewer(s) ask to do so.

** L92 (related to first point) authors use watershed to improve their segmentation workflow. WS could be stated in the introduction.*

As stated in the answer to first comment, we added a sentence l.21-23 to mention the watershed approach as a traditional way to segment nuclei.

** L116: It is not very clear to me why the authors use two different modalities. Is this to increase the genericiy of the segmentation approach? the strategy should be better explained.*

In the biological study, images were acquired with two different modalities, without a real biological meaning but because of technical constraints. However, we use this constraint to have an idea about the genericity of the deep learning approaches. We emphasized this point when comparing the 5 different methods in the first section in l.130-192.

** L118 better justify relevancy of transfer learning (and of using both confocal and wide-field). If the structures to segment are not the same, it is rather surprising to apply the same network. Also, it is not natural to discover the use of transfer learning at his point, this could have been stated earlier.*

We thank Reviewer #2 for this valuable advice. The use of transfer learning is described in the Methods, it helps the Mask R-CNN to converge more quickly to a plateau, even though it only slightly changes results when confocal and widefield images are pooled together, which is not the case otherwise. We added two sentences about this l.176-182. In addition, we added Table S2 to show in one place the pre-processing, normalization, optimization method, use of data augmentation, use of transfer learning and post-processing for each deep learning method used for nuclei segmentation.

** L159: sentence is not clear.*

** L160: this section would benefit from a better explanation of what do the author want to obtain from the image, including an explanation / a recall about the different patterns (punctate or diffuse) that can be observed during cycle, and eventually the associated segmentation difficulties.*

We acknowledge Reviewer #2 for this important point. We changed the beginning of this section to add more information about E2Fs markers as well as EdU and pH3 and directly addressed what we intend to do (l.195-201).

** L160: I understand that thresholding may be limited for segmentation of diffuse patterns. But many image segmentation methods exist and could have been explored. It would be interesting to better explain the difficulty encountered, and better justify why DL seems to be the most adequate.*

The two main difficulties when using a thresholding approach for identifying the markers were to correctly discriminate between dense and punctate patterns for pH3 and particularly EdU, especially with widefield images, and to separate signal in the nuclei and signal in the cytoplasm for E2F4. We emphasized those difficulties in the manuscript to better justify the use of deep learning for marker identification.

** L177: it is not clear if the "Annotater" plugin is a side-product of the research, or if the whole workflow rely on it. This could be clarified.*

Annotater is actually a side-product of the research which has been helpful to manually annotate from scratch and to manually correct nuclei segmentation and marker identification, as well as the identification of markers with an intensity thresholding. We changed the text in the manuscript to better reflect this role for Annotater.

** L204: maybe recall why it is interesting to focus on protein concentration. the transition from previous paragraph is abrupt.*

We changed the transition by introducing other ways to tackle the estimation of protein concentration over the cell cycle, which are not suited to our data (l.246-248).

** L 236: concentrations are not parabola... The representation of their evolution may depict a parabola, however.*

We thank Reviewer #2 for this valuable advice, we corrected this mistake all over the manuscript.

** L360-367: the note at the nd of the paragraph would be more appropriate in the result or in the discussion section.*

We changed the normalization used when training and processing U-Net. This made the U-Net approach less sensitive to data augmentation so that we could use the same data augmentation for both modalities.

** L492-615: this section if quite technical, and it is rather surprising to have such an inbalance between the method description and the exploitation of the results. I wonder if this modelling part can not be an article of itself?*

In the third section, we added an extensive evaluation with simulated data. Mainly, we assessed the impact of the number of samples, the number of intensity and time bins in the presence of noisy data. Finally, we added a new section to test if the temporal evolution of protein concentration can be estimated without marker identification, which also allowed us to evaluate the influence of the training dataset used for nuclei segmentation with respect to the temporal evolution of protein concentration over the cell cycle. With these additions, we feel that the manuscript is better balanced and that the modelling part is better emphasized.

Reviewer #3: This manuscript by T. Pecot and al. propose a workflow, based on deep learning (DL) segmentation and modeling, to reconstruct time courses of protein accumulation from fixed images. This work is timely and important for two reasons. First, while DL has been shown to work well for biological images, most reported uses consider proof of principle, standard datasets, or simple cases; reporting usage in full fledged, biologically relevant, and non trivial example is important and useful. Second in vivo video microscopy is costly and difficult, when it is possible, and recovering time courses from fixed samples in a reliable and efficient way is also important and useful. Overall the paper is well written, the study is well conducted, and the methods used, evaluations done and results reported are convincing.

However, it seem to me to lack a clear purpose, by attempting to be too many things at once and not quite succeeding properly at any of them. Is it an evaluation of standard deep learning architecture for nucleus segmentation in non trivial images? a report on active/interactive learning - human in the loop workflow to try and address the labeling issue for biological images, by introducing a new imagej plugin? or really the presentation of a new workflow of time course estimation from fixed image ? According to the title (and indeed, in my humble opinion as well, since it is the most novel and innovative) it should be the later; however it is the least investigated and evaluated part, as opposed to DL, with one unique graph in the main paper and very little discussion. Suggestions could be:

- to study the influence of DL segmentation accuracy for time course estimation, providing evaluation of the workflow as a whole

We thank Reviewer #3 for this very good idea. We actually added a new section to test if the temporal evolution of protein concentration can be estimated without marker identification. It allowed us to then evaluate the influence of the training dataset used for nuclei segmentation with respect to the temporal evolution of protein concentration over the cell cycle, providing an evaluation of the workflow as a whole.

- additional evaluation of the estimated time courses. Depending on the availability/feasibility of experiments, those could be done with simulation. At the very least corroboration of the found time courses from other experimental or biological arguments.

We acknowledge Reviewer #3 for this important point. We added an extensive evaluation with simulated data. Mainly, we assessed the impact of the number of samples, the number of intensity and time bins on the estimation of protein concentration over the cell cycle, with different levels of noise corruption.

- application of the same technique on other published data

Obviously, applying our approach on other published data and comparing the results with those obtained in the corresponding manuscript would have been a great way to convince people about the importance of our method. Unfortunately, we could not find other studies aiming at the same goal with similar data.

- provide some novel understanding or biological findings those data/methods allowed

Our approach is the first one to show with such temporal precision the succession of E2Fs waves over the cell cycle in living tissue, unlike previous studies that were conducted in cell culture. We feel that the conclusion of Section 3 in itself is a demonstration of the value of this method.

- discuss some of the techniques that has been proposed for similar ends (https://doi.org/10.1038/nmeth.3545 or https://doi.org/10.1038/s41467-017-00623-3 comes to mind for example).

The techniques mentioned by reviewer #3 are very interesting and have proved to be efficient for estimating cell cycle progression of cells. Consequently, we added a reference to them [32,33] to show that this is an important field and that research has been conducted to answer these questions (l.246-248). Unfortunately, these are machine learning methods and depend on data annotation to be trained. This is not possible in our study.

Other points:

- the DL methods are not compared with current/recent SOTA, like cellpose or stardist. Reporting their use (and potentially failure) would be interesting to the community

This is an important point and we added both Cellpose and Stardist in the evaluation of nuclei segmentation with deep learning approaches.

- The efficacy of deep learning for segmenting and classifying biological images is now well accepted and it may not be needed to insist on it to much

We acknowledge that we probably over-emphasized about it, we modified the manuscript by keeping this remark in mind.

- The article talks about 'a small training dataset' but still many tens of images had to be manually segmented. Maybe a study of the influence of training dataset size on accuracy could be interesting, and/or a comparison of

We changed the evaluation of nuclei segmentation and considered 4 different training datasets to evaluate the influence of training dataset size and imaging modalities: 1) confocal images, 2) half of widefield images, 3) all widefield images, 4) confocal and widefield images. We added Table S3 to summarize the number of images and the number of nuclei for each of these training datasets.

- The assumption that "fluorescence intensity is proportional to protein concentration" could be discussed further, since it is not so obvious in IF. [30] seem to be about a novel technique for in vivo expression, it is not clear how it relates to that claim.

We agree with the reviewer that this reference is not entirely appropriate to substantiate this claim and apologize for the oversight. In this article, the authors utilize a genetic fluorescent reporter to measure expression of a protein of interest and show that fluorescence intensity is proportional to the number of molecules of the given protein that a cell produces. Even though the fluorescence-based IHC techniques used in our study are centered on a similar principle (the level of fluorescence derived from the detection systems is determined by the relative abundance of the target antigen, or in other words the level of protein expression), the techniques are not the same. Therefore, we have now included more appropriate references [34,35] that address several basic principles of tissue immunolabeling techniques such as the relevance of the linear dynamic range concerning fluorescence-based IHC techniques for quantitation of protein expression in fixed tissue specimens.

- Maybe a personal preference but bar plot + whiskers may not be the ideal way to report such results. For mean+-standard deviation, simple tables may suffice. Graphical representation could be more efficient when used to display whole distributions, like violin plots or similar techniques.

This remark is in line with one remark of Reviewer #1. We addressed it by only using tables showing mean +- standard deviation in Figure 2 and replaced Figure S5 in the initial submission with Tables S5-6.

- more generally, unless I missed it, it seem the paper has been submitted as a research article and not specifically as a method paper as it is, it may lack novel biological findings for that, but would certainly have its place as a method paper, the comments above notwithstanding.

This is completely true, our mistake.
