# Supplementary material for: Deep learning tools and modeling to estimate the temporal expression of cell cycle proteins from 2D still images
Source: PLoS Comput Biol. 2022 Mar 14;18(3):e1009949. doi: 10.1371/journal.pcbi.1009949 (PMC8947602; doi:10.1371/journal.pcbi.1009949)
Supplement: Attachment — Submitted filename: AnswersToReviewers.pdf [file pcbi.1009949.s026.pdf]

Reviewer #1:

- I thank the authors for taking the time to review my feedback. I found tables S2 and S4 quite helpful to understand the different training pipelines. I would like to see a similar table when comparing the performance of the different methods: I would suggest a given IoU score (or, multiple) - and then comparing performance of all the different methods and showing those numbers in a table. Having to jump between tens of different subfigures to see the impact of data augmentation is very difficult and time consuming. I understand that a table cannot summarize all the results across the different IoU thresholds, and the authors can keep the figures in the supplementary material if necessary, but, it's really necessary to have a single table with the important performance metrics.

We acknowledge that comparing the five deep learning approaches for nuclei segmentation with the different algorithmic variants (data augmentation, transfer learning and post-processing) was laborious and we thank reviewer #1 for this valuable advice. We added two supplementary tables to show the F1 score obtained with IoU=0.5 and IoU=0.75 when considering all algorithmic variants for each method applied to confocal images (S5 Table) and widefield images (S6 Table).

- Figure 2 and 3 are identical in my copy of the manuscript, and I could not evaluate that portion of the manuscript because I couldn't see the data.

We apologize for this unfortunate mistake. We corrected this error in the new version of the manuscript.

- I still insist that if the authors would like to bin E2F data, they should show the raw histogram data justifying a binning procedure. They have that raw data (they need it to obtain the binned values) - and plotting a histogram, together with the cutoff thresholds should be very easy. I understand that this is standard in their field - but - for someone outside of the field looking to evaluate the manuscript, continuous data getting binned without seeing the raw distributions is confusing.

We understand that this step might look confusing to readers, so we added S7 Fig to illustrate how the E2Fs levels of intensity are defined. These levels depend on the range of average intensity observed in the nuclei for each image to normalize intensity over the images. Consequently, we decided to include two images in this figure: the E2F8 channel of a confocal (S7 Fig **a**) and a widefield (S7 Fig **b**) image. The positive and negative nuclei are overlaid on the E2F8 channel for the two images in S7 Fig **a**-**b**. The histograms for the average E2F8 nuclear intensity observed in all cells and the corresponding E2Fs levels represented with colors are shown in S7 Fig **c**-**d**. While the intensity minimum and maximum for each level of intensity are different for the two images, the level width for a given image is the same for all three levels. It has to be noted that negative nuclei with non-specific intensity can have a nuclear intensity similar to positive nuclei (blue and orange bars). Additionally, we added a small paragraph in the Methods about this figure (l.604-615).

- The suggestions from reviewer 2 about presentation and multiple papers are great. Currently- this is two papers, and one paper might be better suited for a computer vision conference or workshop, while the biological findings and applications might belong here. For example - training curves and detailed comparisons to other methods would be really useful if this was a pure computer vision paper, but, in a biology paper, they are probably overkill, as people are not that interested in evaluating the methods at that level of detail.

While we understand how this study can be seen as two distinct manuscripts (one about nuclei segmentation and marker identification, one about the estimation of the protein concentration over the cell cycle), we believe that it is important to present the workflow in its entirety to assess the validity of the approach as nuclei segmentation and marker identification are impacting the final result. Adding the fourth section in which we propose to estimate the protein concentration evolution over the cell cycle without marker identification allowed us to also assess the influence of the training datasets used for nuclei segmentation on the final estimation, providing an evaluation of the workflow as a whole. We believe that it justifies putting together all this study.

Reviewer #3: The resubmitted article is much improved compared to the first version and in particular more focused. I have no major request of changes to be made before publication, just a few comments below.

The figures are wrong in the submitted manuscript (fig 2 and 3 are the same and Fig 4 seem unrelated). A bit problematic for the review but the main novelty are the simulations, which are in the supplementary. To be thoroughly checked before publication though...

We apologize for this unfortunate mistake. We corrected this error in the new version of the manuscript.

Fig 3 has only the final results with all simulation and method detail relegated in the supplementary. I personally think it's a bit of a shame since the figures are the main points of entry for many reader, which would make that work invisible. I would welcome some of that in the main figures, but leave that choice to the authors/editors.

We thank Reviewer #3 for this valuable advice. We added Figure 4 that summarizes the evaluation with simulations when considering the same parameters (number of samples, intensity and time bins) than those used for real data with a proportion of bins corrupted with noise ranging from 0% to 50%.

The simulations have pretty big error bars, and the noise seem to be only marginally affecting the results in many cases with averages being fairly constant from 0 to 30 and often 50% noise. Can the authors comment on that? Make one wonder if that is the most informative way of displaying that result, and what would the error bar on real data would be. Also the simulations are presented in the text before the time course estimation methods, while it uses/is meant to test it, which is a bit strange.

We acknowledge that presenting the simulations before the real data while they were designed to evaluate the approach on real data was misleading. Consequently, we moved the section on simulations at the end of Section 3 (l.309-330). Then, we decided to use the same scale for the MSE for each figure (S12-S14 Figs) to make comparisons easier between E2Fs, EdU and pH3. We also removed the evaluation with 20 bins for intensity (S14 Fig) as it was masking the other results for a lower number of bins. It appears that error bars are really large for situations where the parameters (number of samples or number of bins) are not suited. Otherwise, error bars do not look unreasonably large, and MSE increases with the proportion of bins corrupted with noise. Finally, we agree that this might not be a great way to display the results, but we were not able to find a more suited way to display all the information residing in these graphs.
