# Supplementary material for: Deep learning tools and modeling to estimate the temporal expression of cell cycle proteins from 2D still images
Source: PLoS Comput Biol. 2022 Mar 14;18(3):e1009949. doi: 10.1371/journal.pcbi.1009949 (PMC8947602; doi:10.1371/journal.pcbi.1009949)
Supplement: Attachment — Submitted filename: AnswersToReviewers.pdf [file pcbi.1009949.s027.pdf]

Reviewer #1:

I thank the authors for all the additions since the last review. I found the extra tables especially helpful, and appreciated the additional figure showing the E2F concentration.

In my view, the E2F quantization step is not justified on the basis of data: the distribution in figure S7 is obviously not multi-modal, and, in the material and methods section, the thresholding step was described as using 'manually defined thresholds', and there's no discussion at all about how those thresholds were calculated or how sensitive the results later in the paper are to the choice of this arbitrary threshold.

It might be that there is a biological justification for this thresholding step - and - at the very least, I'd like the authors discuss this in more detail in the main text. Currently, the main text has a quote:

"We assume that fluorescence intensity is proportional to protein =concentration [34, 35] and therefore define quantized levels of intensity for E2Fs."

Clearly, the second part (deciding to quantize a signal) does not follow from the first (protein concentration being proportional to the fluorescent signal).

We acknowledge that the definition of negative and positive cells for E2Fs quantization was not explicitly explained, potentially confusing the reader and we thank the reviewer for this valuable remark.

As demonstrated in [34, 35], fluorescence intensity is proportional to protein concentration. Consequently, high intensity corresponds to high protein concentration while low intensity corresponds to low intensity concentration. Quantizing intensity reduces the number of intensity bins, but there is still correspondence between high/low protein concentration and high/low intensity. Then, a high number of intensity bins negatively impacts the estimation of E2Fs concentration over the cell cycle as shown in Figure S14 when considering 10 bins. Finally, scoring of immuno-staining according to intensity into four bins is common practice in both diagnostic pathology and biomedical research [36, 37].

While not explicitly described in the previous version of our manuscript, there is no thresholding involved to quantize E2Fs levels. Actually, positive and negative cells were manually selected to define the training and evaluation datasets in the section about marker identification. Consequently, negative cells are known and assigned an intensity equal to 0. Then, for each individual image, the average fluorescence intensity for each positive cell is measured and the range of average intensities from the lowest to the highest is binned into three to define levels 1 to 3. This intensity assignment amounts to normalizing intensity over the images. As some cells show non-specific fluorescence, a few negative cells show similar average nuclear intensity (blue and orange bars in Figure S7 **c-d**) than positive cells. This demonstrates the advantage of identifying positive and negative cells without an intensity thresholding-based method. This also explains why the Inception-V3 approach is superior to manual thresholding for marker identification as shown in Figure 2. The manuscript has been modified to clearly explain how E2Fs quantization is conducted (l.596-603).
